# Online Instruction: a practical guide for librarians

**DOI:** 10.29173/jchla29671

**Published:** 2023-04-01

**Authors:** Melanie Anderson

**Affiliations:** University Health Network Toronto, ON, Canada

Mroczek E. **Online Instruction: a practical guide for librarians**. Lanham, Maryland: Rowman & Littlefield; 2022. Paperback: 137 p. ISBN 9781538157671. Price: USD$65. Available from: https://rowman.com/ISBN/9781538157671/Online-Instruction-A-Practical-Guide-for-Librarians.

Early in the pandemic library staff everywhere had to choose if and how to move in-person instruction and programming online. For some, online instruction was familiar, for others, it was a brand-new world. It has become clear that online programming and instruction are not going away. In this context, Emily Mroczek, a children’s librarian in Chicago with a background in instructional design and instruction, wrote *Online instruction: a practical guide for librarians*.

*Online Instruction* is an introductory level guide covering the basics of online instruction, including different methods of offering instruction, types of programming and instruction that could be adapted to online delivery, and tools that could make online instruction engaging and effective.

With less than 150 pages and only eleven chapters, coverage of any one topic is necessarily limited. Readers wishing for greater depth of coverage will need to seek other sources. The book is organized for readers to jump straight into any chapter that addresses their immediate questions, with no need to read the book through from beginning to end.

Chapters address topics ranging from “building community”, “evaluating and recommending resources”, using Zoom, copyright issues, and digital accessibility. The contents of each chapter include explanations of terminology; lists of tips, ideas, and resources that readers could employ; a variety of tables and images; and brief stories from librarians doing online programming or instruction related to the topic of the chapter. Each chapter has a list of references, and there is a bibliography at the end for readers looking for more information.

It is important to note that all laws, regulations, and legal requirements mentioned in the book are specific to the United States. Canadian readers will need to turn to local sources for information about copyright, accessibility standards and requirements, privacy, etc. However, there are also suggested resources for copyright-free or open images and accessibility tools that everyone should be able to use. Given the broad audience intended for this book, no one will be surprised to find that most examples and hypothetical situations are based in public, college, and university libraries, and may be difficult to transfer into some hospital, professional organization, research institute, or other special library settings.

There are several other recent books about aspects of online instruction in libraries, however, most appear to be aimed at a more specific audience or to discuss more advanced concepts. This text is both aimed at and best used by beginners in the world of online instruction. Readers with either minimal experience or training with online instruction, or those who have been restricted to offering online presentations and want to branch out, may find the definitions, tips, and tool suggestions helpful, and the tone friendly and accessible.

Readers looking for a “how to” guide may find themselves disappointed. Understandably, considering the broad audience the text is aiming to serve, Mroczek has offered information, suggestions, tips, and ideas, but not step-by-step instructions. For those looking for a “how to”, the final chapter, Putting it all together, does offer a list of “actionable steps” grouped by professional roles. While not step by step instructions, these recommendations refer to the contents of earlier chapters and are recommended as places to start if readers are overwhelmed by the possibilities for online instruction.

For some of the more abstract or complex topics, readers are likely to find the information provided difficult to apply to their own practices without seeking out more sources, advice, or training. For instance the eight-page second chapter, Pedagogical matters, defines pedagogy and why it is important; notes that it is best to place pedagogy before technology; lists and provides some information about popular teaching models; lists and discusses digital pedagogies including discussing the Association of College and Research Libraries’ *Standards for proficiencies for instruction librarians and coordinators* (2007); defines and discusses types of presence and relationships as part of instruction; and highlights the importance of communication, engagement, and active learning. All of this is important information. However, neither the self reflection questions at the end of the chapter nor the sections discussing teaching models provide guidance to the reader about what a teaching practice would or would not look like when guided by one or more of the models. A reader interested but inexperienced in constructivism or Bloom’s Taxonomy who decides they want to use it as part of their instruction practice would need to seek out both additional information and practical advice about how it can or should be applied in their circumstances.

Other areas lend themselves more directly to application, such as the information and question prompts in the Choosing a platform chapter, in which readers can easily eliminate platforms based on their own circumstances and needs. Unfortunately, just because of the nature of online apps and resources, this information will become dated quickly. That said, the questions people need to consider as they choose platforms should remain relevant.

In summary, *Online Instruction: a practical guide for librarians* is a short, accessible, and friendly first step for those starting out with online instruction or programming. Canadian audiences, particularly those in special library settings, may find that portions of the book are not relevant to their circumstances. Those who are already somewhat familiar with some of the terminology and practices around online instruction can find some specific tools to try out, or possibly new ideas for program delivery, but otherwise may prefer more advanced texts or participation in continuing education opportunities instead.

